# Points for Trained Nurses

**Published:** 1894-04

**Authors:** 


POINTS FOR TRAINED NURSES.—At the training schools for nurses no applicants are accepted who are under twenty-one years of age or over thirty-five; twenty-five is the preferred age. When application is made by letter it must be addressed to the superintendent of the school. In reply she will receive a circular stating that a personal interview is desirable. If that is impossible, the applicant should wrice again, saying so and asking for an application blank. This blank must be filled and in the applicant's own handwriting and returned to the superintendent, together with a physician's certificate of health, a letter from a clergyman and the address of three women, not relatives, who have known the applicant for several years. These applications are filed, and when a vacancy occurs the most desirable applicant is selected by the president, and is taken for a month on trial. During this month of probation, she will, at almost all the training schools receive her board and lodging. At the end of the month she may be accepted or rejected as a pupil nurse, and the decision is final.—February *Ladies' Home Journal.*

